# Protocol to identify defined reprogramming factor expression using a factor-indexing single-nuclei multiome sequencing approach

**DOI:** 10.1016/j.xpro.2024.103148

**Published:** 2024-06-22

**Authors:** Liangru Fei, Kaiyang Zhang, Sampsa Hautaniemi, Biswajyoti Sahu

**Affiliations:** 1 Centre for Molecular Medicine Norway, Faculty of Medicine, University of Oslo, Gaustadelléen 21, 0349 Oslo, Norway; 2 Research Program in Systems Oncology, Research Programs Unit, Faculty of Medicine, University of Helsinki, Haartmaninkatu 8, 00014 Helsinki, Finland; 3 Applied Tumor Genomics Program, Research Programs Unit, Faculty of Medicine, University of Helsinki, Haartmaninkatu 8, 00014 Helsinki, Finland; 4 iCAN Digital Precision Cancer Medicine Flagship, University of Helsinki, Haartmaninkatu 8, 00014 Helsinki, Finland; 5 Department of Cancer Genetics, Institute for Cancer Research, Oslo University Hospital, Oslo, Norway

**Keywords:** Genomics, Sequencing, Molecular Biology

## Abstract

Ectopic expression of lineage-specific transcription factors (TFs) of another cell type can induce cell fate reprogramming. However, the heterogeneity of reprogramming cells has been a challenge for data interpretation and model evaluation. Here, we present a protocol to characterize cells expressing defined factors during direct cell reprogramming using a factor-indexing approach based on single-nuclei multiome sequencing (FI-snMultiome-seq). We describe the steps for barcoding TFs, converting human fibroblasts to pancreatic ductal-like cells using defined TFs, and preparing library for FI-snMultiome-seq analysis.

For complete details on the use and execution of this protocol, please refer to Fei et al.^1^

## Highlights

• Generating barcoded Gateway destination vectors by PCR
• Barcoding individual transcription factor constructs by Gateway cloning
• Direct reprogramming of pancreatic ductal-like cells from human fibroblasts
• Characterizing the cells expressing defined TFs in a heterogeneous cell population

## Abstract (editor highlights)

Publisher’s note: Undertaking any experimental protocol requires adherence to local institutional guidelines for laboratory safety and ethics.

## Graphical abstract

**Figure:**
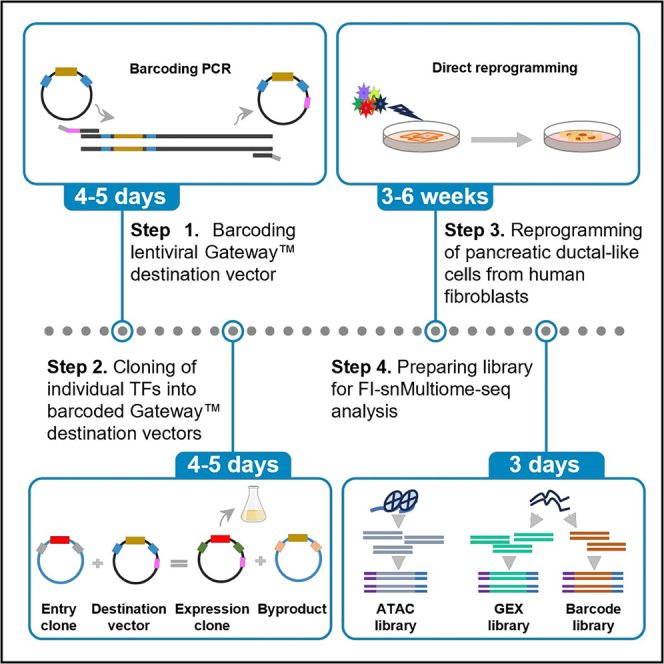

## Before you begin

This protocol below describes the steps to generate barcoded TF constructs for inducing pancreatic ductal-like cells from human fibroblasts and for further use with FI-snMultiome-seq analysis. Using this protocol, we identified the cells carrying different combinations of reprogramming TFs from a six-TF (6F) pool and compared the enrichment of ductal cell signatures in each clone. We demonstrated that all six TFs are necessary and sufficient for efficient conversion of pancreatic ductal-like cells from human fibroblasts.^1^ Gene expression and chromatin profiling of the cells using FI-snMultiome-seq assay provided a high-resolution single-nucleus analysis of TF-mediated reprogramming during transdifferentiation.

The protocol is versatile and can be adapted to other direct reprogramming models and experimental settings that need to characterize defined cells expressing the gene(s) of interest from exogeneous vectors in a heterogeneous cell population.

Before starting, you need to identify candidate TFs required for your reprogramming model or the gene(s) of interest for your experimental setting, and clone the full-length open reading frames (ORF) of all individual TFs/genes into Gateway donor vectors. Also, verify to have all the reagents and equivalent equipment described in the protocol.

### Institutional permissions

Experiments on live vertebrates or higher invertebrates must be performed in accordance with national guidelines and regulations. Experiments involving lentivirus must be conducted under Biosafety Level 2 (BSL2) as per institutional guidelines. We remind readers to obtain all the necessary permissions from the relevant institutions before starting the experiment.

### Identify candidate TFs for your reprogramming model

**Timing: variable**

1. Select candidate TFs based on their reported role in developmental biology and/or use computational framework, such as Mogrify (https://mogrify.net/index),^2^ to predict candidate TFs required for transcriptomic switches from your source cell type to any target cell type.

***Note:*** Computational frameworks based on gene expression data and regulatory network information can usually predict the TFs required for cell fate conversion for broad tissue types instead of specific cell types.^2^^,^^3^ You may combine the literature-curated TFs and computational framework-predicted TFs as initial pool to start with.

### Clone TF ORFs into Gateway donor vectors to generate entry clones

**Timing: 1–2 weeks**

2. Obtain the full-length ORFs of all individual TFs on Gateway donor vectors from publicly available resources such as Addgene or get the *attB* flanked TF ORFs from any commercial source and clone into Gateway donor vector (e.g., pDNOR221) by BP Reaction following manufacturer’s instructions.

## Key resources table

**Table:** 

REAGENT or RESOURCE	SOURCE	IDENTIFIER
**Bacterial and virus strains**		

One Shot Stbl3 chemically competent *E. coli*	Thermo Fisher Scientific	Cat# C737303
One Shot *ccd*B Survival 2 T1R competent cells	Thermo Fisher Scientific	Cat# A10460
pLenti6/V5-Barcode-FOXA2	Fei et al.^1^	N/A
pLenti6/V5-Barcode-HNF1B	Fei et al.^1^	N/A
pLenti6/V5-Barcode-HNF6	Fei et al.^1^	N/A
pLenti6/V5-Barcode-PDX1	Fei et al.^1^	N/A
pLenti6/V5-Barcode-SOX9	Fei et al.^1^	N/A
pLenti6/V5-Barcode-SOX17	Fei et al.^1^	N/A

**Chemicals, peptides, and recombinant proteins**		

Ascorbic Acid	Sigma-Aldrich	Cat# A1300000
Retinoic Acid	Sigma-Aldrich	Cat# R2625
PD0325901	Sigma-Aldrich	Cat# PZ0162
Poly-L-lysine	Sigma-Aldrich	Cat# P4707
Polybrene	Sigma-Aldrich	Cat# S2667
CHIR99021	StemMACS	Cat# 130-106-539
LDN193189	StemMACS	Cat# 130-103-925
Activin A	PeproTech	Cat# AF-120-14E
FGF7	PeproTech	Cat# AF-100-19
Complete human epithelial cell medium	Cell Biologics	Cat# PB-H-6621
SPRIselect beads	Beckman Coulter	Cat# B23318
Electrophoresis gels, peqGOLD, universal agarose	VWR	Cat# 732-2789P
Buffer EB	QIAGEN	Cat# 19086
Lenti-X concentrator	Takara	Cat# 631232
Matrigel	Corning	Cat# 356230
Q5 high-fidelity DNA polymerase	New England Biolabs	Cat# M0491
KAPA HiFi HotStart ReadyMix	Kapa Biosystems	Cat# KK2602
dNTP mix	Thermo Fisher Scientific	Cat# R0192
SYBR Green I nucleic acid gel stain	Thermo Fisher Scientific	Cat# S7563
Nuclease-free water	Thermo Fisher Scientific	Cat# AM9937
FastDigest NheI	Thermo Fisher Scientific	Cat# FD0974
FastDigest Dpnl	Thermo Fisher Scientific	Cat# FD1703
FastDigest XhoI	Thermo Fisher Scientific	Cat# FD0694
FastDigest AflII	Thermo Fisher Scientific	Cat# FD0834
T4 DNA ligase	Thermo Fisher Scientific	Cat# EL0011
Gateway LR Clonase II enzyme mix	Thermo Fisher Scientific	Cat# 11791020
Lipofectamine 2000 transfection reagent	Thermo Fisher Scientific	Cat# 11668019
Geneticin selective antibiotic (G418 sulfate)	Thermo Fisher Scientific	Cat# 10131027
GlutaMAX supplement	Thermo Fisher Scientific	Cat# 35050038
B27 supplement	Thermo Fisher Scientific	Cat# 17504044
Sodium pyruvate	Thermo Fisher Scientific	Cat# 11360070
L-glutamine	Thermo Fisher Scientific	Cat# 25030024
MEM non-essential amino acids (MEM NEAAs)	Thermo Fisher Scientific	Cat# 11140035
Penicillin-streptomycin (Pen-Strep)	Thermo Fisher Scientific	Cat# 15140122
2-mercaptoethanol	Thermo Fisher Scientific	Cat# 21985023
HEPES	Thermo Fisher Scientific	Cat# 15630080
Insulin-transferrin-selenium-ethanolamine (ITS -X)	Thermo Fisher Scientific	Cat# 51500056
KnockOut serum replacement	Thermo Fisher Scientific	Cat# 10828010
Fetal bovine serum (FBS)	Thermo Fisher Scientific	Cat# 10270106
StemPro Accutase cell dissociation reagent	Thermo Fisher Scientific	Cat# A1110501
Opti-MEM I reduced serum medium (Opti-MEM)	Thermo Fisher Scientific	Cat# 51985034
Advanced DMEM/F-12	Thermo Fisher Scientific	Cat# 12634010
DMEM, high glucose, no glutamine	Thermo Fisher Scientific	Cat# 11960085
DPBS, no calcium, no magnesium	Thermo Fisher Scientific	Cat# 14190250

**Critical commercial assays**		

Chromium next GEM single cell multiome ATAC + gene expression reagent bundle	10× Genomics	Cat# PN-1000285
Dual index kit TT set A	10× Genomics	Cat# PN-100021
NucleoSpin gel and PCR clean-up, mini kit for gel extraction and PCR clean-up	MACHEREY-NAGEL	Cat# 740609
NucleoSpin plasmid, mini kit for plasmid DNA	MACHEREY-NAGEL	Cat# 740588
NucleoBond Xtra midi EF, midi kit for endotoxin-free plasmid DNA	MACHEREY-NAGEL	Cat# 740420
KAPA library quantification kit	Kapa Biosystems	Cat# KK4854
Agilent high sensitivity D5000 ScreenTape	Agilent	Cat# 5067-5592
Agilent high sensitivity D5000 reagents	Agilent	Cat# 5067-5593

**Deposited data**		

Raw data	Fei et al.^1^	GEO: GSE216859
ENCODE blacklisted genomic regions for hg38	ENCODE	ENCFF356LFX
Human reference genome NCBI build 38, GRCh38	Genome Reference Consortium	https://www.ncbi.nlm.nih.gov/assembly/GCF_000001405

**Experimental models: Cell lines**		

Human foreskin fibroblasts (HFF)	ATCC	Cat# CRL-2429
293FT cell line	Thermo Fisher Scientific	Cat# R70007

**Oligonucleotides**		

Barcode_Temp_Nhel_Forward primer: CATGCTA GCNNNNNNNNNNNNNNNNNNNNCGAGCTC GGTACCTTTAAGACC	Fei et al.^1^	N/A
Barcode_Temp_Nhel_Reverse primer: CATGCTA GCTTGTGCTTAGCCCTCCCACAC	Fei et al.^1^	N/A
Sseq_ORF sequencing primer: CCAGTGTGGTGG AATTCTGCA	Fei et al.^1^	N/A
Sseq_Barcode sequencing primer: GCTGCAATAAA CAAGTTCCTCTCAC	Fei et al.^1^	N/A
Truseq_Read2_Vec_Amp_Forward primer: GTGAC TGGAGTTCAGACGTGTGCTCTTCCGATCTAGGG CTAAGCACAAGCTAG∗C	Fei et al.^1^	N/A
Truseq_Read1_Amp_Reverse primer: ACACTCTTTC CCTACACGACGCTCTTCCGATC∗T	Fei et al.^1^	N/A

**Recombinant DNA**		

psPAX2	Addgene	RRID: Addgene_12260
pMD2.G	Addgene	RRID: Addgene_12259
pDONR221 vector	Thermo Fisher Scientific	Cat# 12536017
pLenti6/V5-DEST	Thermo Fisher Scientific	Cat# V49610

**Software and algorithms**		

SegIO	Roberts et al.^4^	https://github.com/google/seqio
Biopython	Cock et al.^5^	https://biopython.org/
Seurat (v4.1.1)	Hao et al.^6^	https://satijalab.org/seurat/
Signac (v1.7.0)	Stuart et al.^7^	https://stuartlab.org/signac/
MACS2 (v2.2.7.1)	Zhang et al.^8^	http://github.com/taoliu/MACS/
chromVAR (v1.18.0)	Schep et al.^9^	https://stuartlab.org/signac/reference/runchromvar
ScType (v1.0)	Ianevski et al.^10^	https://github.com/IanevskiAleksandr/sc-type

**Other**		

Millipore Steriflip vacuum tube top filter	Sigma	Cat# SE1M003M00
10× magnetic separator	10× Genomics	Cat# 120250
Agilent TapeStation 4150 system	Agilent	Cat# G2992AA
LightCycler 480	Roche	Cat# 05015278001
C1000 Touch thermal cycler	Bio-Rad	Cat# 1851197
ThermoMixer	Eppendorf	Cat# 2231001005

## Materials and equipment

**Table:** Complete medium for 293FT cells

Reagent	Final concentration	Amount
MEM NEAAs (100×)	1×	5 mL
L-Glutamine (200 mM)	6 mM	15 mL
Sodium Pyruvate (100 mM)	1 mM	5 mL
FBS	10%	50 mL
DMEM	N/A	425 mL
**Total**	**N/A**	**500 mL**

Store at 4°C for up to 1 month.

**Table:** Serum reduced medium for 293FT cells

Reagent	Final concentration	Amount
FBS	5%	12.5 mL
Opti-MEM	N/A	237.5 mL
**Total**	**N/A**	**250 mL**

Store at 4°C for up to 1 month.

**Table:** Complete medium for fibroblasts

Reagent	Final concentration	Amount
Pen-Strep (10,000 U/mL)	100 U/mL	5 mL
L-Glutamine (200 mM)	2 mM	5 mL
Sodium Pyruvate (100 mM)	1 mM	5 mL
FBS	10%	50 mL
DMEM	N/A	435 mL
**Total**	**N/A**	**500 mL**

Store at 4°C for up to 1 month.

**Table:** Basal medium for reprogramming cells

Reagent	Final concentration	Amount
Ascorbic Acid (500 μg/μL)	50 μg/mL	5 μL
B27 Supplement (50×)	1×	1 mL
GlutaMAX Supplement (100×)	1×	500 μL
Pen-Strep (10,000 U/mL)	100 U/mL	500 μL
HEPES (1 M)	25 mM	1.25 mL
2-Mercaptoethanol (55 mM)	0.11 mM	100 μL
ITS-X (100×)	0.5×	250 μL
KnockOut Serum Replacement	1%	500 μL
DMEM/F12	N/A	45.895 mL
**Total**	**N/A**	**50 mL**

Protect from light and store at 4°C for up to 2 weeks.

**CRITICAL:** 2-Mercaptoethanol is toxic if swallowed or inhaled. It causes serious eye damage and skin irritation. Wear personal protective equipment and use only under a chemical fume hood.

**Table:** Maintenance medium for reprogramming cells

Reagent	Final concentration	Amount
Ascorbic acid (500 μg/μL)	50 μg/mL	5 μL
GlutaMAX Supplement (100×)	1×	500 μL
KnockOut Serum Replacement	5%	2.5 mL
Complete Human Epithelial Cell Medium W/O FBS	N/A	46.995 mL
**Total**	**N/A**	**50 mL**

Protect from light and store at 4°C for up to 2 weeks.

## Step-by-step method details

### Barcoding lentiviral Gateway destination vector

**Timing: 4–5 days**

This section aims to generate barcoded pLenti6/V5-DEST vectors.

1. Barcoding pLenti6/V5-DEST vector by PCR and gel extraction of PCR product:
  a. Prepare the PCR reaction master mix on ice as described in the table below.
    **Table:** PCR reaction master mix

Reagent	Amount
Circular pLenti6/V5-DEST Vector	10 ng
Q5 High-Fidelity DNA Polymerase	0.5 μL
10 μM Barcode_Temp_Nhel_Forward Primer	2.5 μL
10 μM Barcode_Temp_Nhel_Reverse Primer	2.5 μL
5× Q5 Reaction Buffer	10 μL
10 mM dNTP Mix	1 μL
Nuclease-Free Water	to 50 μL

    ***Note:*** 10× Multiome Gel Beads include a poly(dT) sequence that enables capture of 3′ poly-adenylated mRNA for gene expression (GEX) library. It cannot detect transcripts originating from exogeneous vectors with >1 kb distance between polyA and the ORF. This barcoding PCR reaction will introduce a 20-bp random oligo (N20) with NheI site into pLenti6/V5-DEST vector downstream of the ORF and 78 bp upstream of its 3′ LTR region, which contains the polyA. Inserting TF barcodes close to polyA enables their efficient capture and optimal library size for Illumina sequencing. One barcoding PCR reaction will generate a complex pool of barcoded vectors that can be used to barcode as many TFs as needed and the excess PCR product can be kept for future use. Designing individual barcode primers for barcoding PCR to avoid screening uniquely barcoded vectors is also feasible when working with less than three TFs.
    **CRITICAL:** Prepare 4–6 reactions to obtain enough PCR product after clean-up and to maintain its complexity.
  b. Gently mix the reaction by pipetting and centrifuge briefly. Start PCR using the cycling conditions described in the table below.
    **Table:** PCR cycling conditions

Steps	Temperature	Time	Cycles
Initial Denaturation	98°C	30 s	1
Denaturation	98°C	10 s	30 cycles
Annealing and Extension	72°C	5 min	
Final extension	72°C	10 min	1
Hold	4°C	∞
  c. Load PCR product on a 0.7% agarose gel and run gel at 120 V for 1 h. Expected PCR product, linear pLenti6/V5-DEST-Barcode vector with Nhel site at both ends, is 8,724 bp (Figure 1A). Gel-purify the PCR product following manufacturer’s instruction. Troubleshooting 1.
    ***Note:*** DNA fragments may exhibit a slight shift of towards higher molecular weight on agarose gel before clean-up (Figure 1A).
    **Pause point:** Store purified product at −20°C for long-term storage or proceed to the next step.

**Figure 1:**
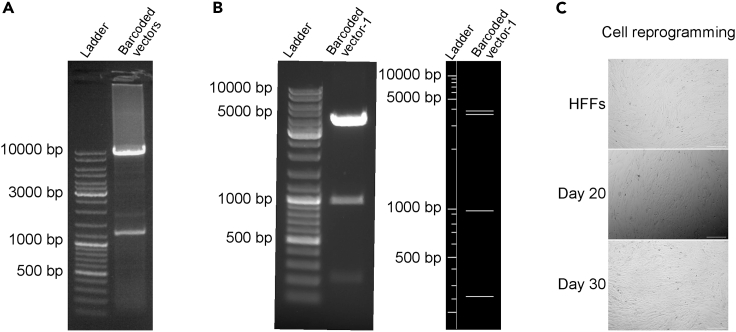
Barcoding PCR product and morphological changes of cells during reprogramming (A) Gel image of barcoding PCR product showing the expected product at 8,724 bp. (B) Gel images of restriction digested barcoded vector pLenti6/V5-DEST vector-1 (left) and the *in silico* digestion using NEBcutter (right). (C) Cell morphological changes during reprogramming. Scale bar represents 100 μm.
2. Restriction digest of linear pLenti6/V5-DEST-Barcode vectors to create sticky ends, self-circularization and transformation:
  a. Prepare following reaction components and mix gently. Spin down quickly and incubate at 37°C for 1 h.
    **Table:** Restriction digest mix

Reagent	Amount
Linear pLenti6/V5-DEST-Barcode Vector	800 ng
FastDigest Buffer (10×)	2 μL
Nhel	1 μL
Dpnl	1 μL
Nuclease-Free Water	to 20 μL

    ***Note:*** Nhel creates sticky ends for self-circularization. Dpnl helps to remove circular template from barcoding PCR reaction in step 1.
  b. Gel-purify the restriction digested product following manufacturer’s instruction.
  c. Prepare following reaction components and mix thoroughly. Spin down quickly and incubate at 16°C for 12 − 20 h.

**Table:** DNA ligation mix

Reagent	Amount
Restriction Digested Linear pLenti6/V5-DEST-Barcode Vector	50 ng
10× T4 DNA Ligase Buffer	5 μL
T4 DNA Ligase	1 μL
Nuclease-Free Water	to 50 μL
  d. Use up to 2 μL of the mixture for transformation of 50 μL *ccd*B Survival 2T1R competent cells. Troubleshooting 1.
    **Pause point:** Store ligated product at −20°C for up to 1 month or proceed to the next step.
3. Screening of pLenti6/V5-DEST vectors carrying unique barcodes:
  a. Screen single colonies and purify plasmids following manufacturer’s instruction.
  b. Verify pLenti6/V5-DEST-Barcode clones by restriction digest and obtain the barcode sequences of individual clones by Sanger sequencing.
    i. Prepare following reaction components and mix gently. Spin down quickly and incubate at 37°C for 1 h.
      **Table:** Restriction digest mix

Reagent	Amount
pLenti6/V5-DEST-Barcode Vector	500 ng
FastDigest Buffer (10×)	2 μL
AflII	1 μL
Xhol	1 μL
Nhel	1 μL
Nuclease-Free Water	to 20 μL

      ***Note:*** AflII and Xhol help to confirm that no rearrangement in the LTR regions of pLenti6/V5-DEST-Barcode vectors has taken place. Nhel helps to ensure the insertion of barcode.
    ii. Run restriction digested product on 0.7% agarose gel. Expected bands from correct clones are 3876 bp, 3656 bp, 960 bp and 220 bp (Figure 1B).
    iii. Obtain the barcode sequence for individual clones by Sanger sequencing using Sseq_Barcode primer (see key resources table). Troubleshooting 2.
      **CRITICAL:** Avoid using the barcodes that introduce new AflII, Xhol and Nhel sites.

### Cloning of individual TFs into barcoded Gateway destination vectors

**Timing: 4–5 days, depends on the number of TFs**

This section is aimed at cloning individual TFs into barcoded pLenti6/V5-DEST vectors and preparing the plasmids of barcoded TFs for lentivirus production.

4. Gateway recombination cloning and plasmid purification:
  a. Clone the ORFs of individual TFs into barcoded pLenti6/V5-DEST vectors by LR reaction following manufacturer’s instruction.
    **CRITICAL:** Use different barcoded pLenti6/V5-DEST vectors for individual TFs to ensure each TF is labeled with a unique barcode.
  b. Use 1 μL of LR reaction for transformation of 50 μL Stbl3 competent cells.
  c. Screen for positive clones by restriction digest as described in step 3b and further verify the sequences of ORF and barcodes by Sanger sequencing using Sseq_ORF and Sseq_Barcode primers (see key resources table), respectively.
  d. Perform plasmid Midiprep for each barcoded factor following manufacturer’s instruction.

### Reprogramming of pancreatic ductal-like cells from human fibroblasts

**Timing: 3–6 weeks, depends on the desired time points for investigation**

This section describes how to induce pancreatic ductal-like cells from human fibroblasts through lentiviral expression of defined TFs.

5. Lentivirus production in 293FT cells:
  Culture 293FT cells as per manufacturer’s instruction.
  **CRITICAL:** Follow biosafety level 2 (BSL2) guidelines while working with lentivirus and be careful with the storage and disposal of biohazard waste.
  a. Poly-L-lysine coating.
    i. Coat T75 flask with 3 mL of 0.001% poly-L-lysine in DPBS. Incubate at 20°C–25°C for 15−20 min with gentle rotation to ensure even distribution.
    ii. Wash twice with DPBS and once with sterile distilled water.
    iii. Air dry for at least 2 h.
      ***Note:*** Coat flasks under sterile hood and store air-dried flasks in sterile conditions at 20°C–25°C for up to 2 weeks.
  b. Day 0: Seed 293FT packaging cells in poly-L-lysine coated T75 flask in complete medium, at 5 × 10^6^ cells per flask.
    ***Note:*** For the maintenance of 293T cells, use complete medium containing 500 μg/mL Geneticin.
  c. Day 1: Change medium to 8 mL of serum reduced medium at 2 h prior to transfection and transfect cells with the combination of transgene plasmid, packaging plasmid psPAX2 and envelope plasmid pMD2.G as follow:
    i. Prepare two 5 mL tubes with following components for one T75 flask of cells and incubate at 20°C–25°C for 5 min.

**Table:** 

Tube	Reagent
Tube 1	48 μL Lipofectamine 2000 in 1 mL Opti-MEM
Tube 2	12 μg transgene plasmid + 9 μg psPAX2 + 3 μg pMD2.G in 1 mL Opti-MEM
    ii. Transfer the reagent in Tube 2 to Tube 1. Mix gently and incubate at 20°C–25°C for 15 min.
    iii. Add transfection mixture dropwise to the cell culture flask, gently swirl to evenly distribute the transfection reagents.
  d. Day 2: Change medium. Add 20 mL of fresh complete medium.
    ***Note:*** Medium change can be performed at 8–16 h after transfection. Long-time transfection will reduce cell viability as the cells are cultured in serum reduced medium at this stage.
  e. Day 4: Harvest virus:
    i. Collect 20 mL of cell supernatant and centrifuge at 500 × *g* for 10 min at 20°C–25°C.
    ii. Filter through 0.45 μm vacuum filters to remove cell debris.
    iii. Add 6.7 mL of Lenti-X concentrator to viral supernatant and mix gently by inverting tube. Incubate at 4°C for 2 h.
    iv. Centrifuge at 1,500 × *g* for 45 min at 4°C.
    v. Remove supernatant and gently resuspend the pellet in 100 μL of DMEM. Store at −80°C in aliquots.
    vi. Titer virus following manufacturer’s instruction. Troubleshooting 3.
      ***Note:*** Viral titers were determined using Perkin Elmer p24 ELISA Kit that represents physical titer based on the concentration of p24 protein: 1 pg/mL of p24 ≈ 10^4^ lentiviral particles/mL ≈ 100 TU/mL, when considering each lentiviral particle contains 2,000 molecules of p24. Of note, physical titer includes free p24 and defective viral particles in addition to the infectious viral particles. To be more accurate, we also performed the functional tittering (TU/mL) by transducing HFFs with pLenti6/V5-EGFP virus, followed by fluorescence tittering protocol for lentivirus from Addgene. For the same pLenti6/V5-EGFP virus preparation, the functional titer approximately corresponds to 1% of the physical titer from p24 ELISA assay. Based on this comparison, the physical titers of other lentiviruses were converted to functional titers for infecting HFFs by diving physical titers with 100. Then MOI was calculated as follows:
      $$\text{MOI}=\frac{\text{Functional}\;\text{titer}\;\text{of}\;\text{virus}\left(\frac{\text{TU}}{\text{mL}}\right)\times \text{Virus}\;\text{volume}\;\left(\mu L\right)}{1000\times \text{Cell}\;\text{number}}$$
6. Matrigel coating:
  a. Thaw matrigel on ice and mix to homogeneity using pre-chilled pipets.
    **CRITICAL:** Matrigel will start to gel above 10°C. Keep matrigel on ice all the time while working. Pre-chill all plastics or media coming in contact with matrigel.
  b. Dilute matrigel in 1:200 using chilled DMEM and add 200 μL of diluted matrigel per well in 24-well plate. Incubate at 20°C–25°C for 1 h.
    ***Note:*** A dilution of 1:200 only results in a thin, non-gelled protein layer which mainly helps with cell attachment.
  c. Remove unbound material and rinse twice using DMEM.
    **Pause point:** If not using the plate immediately, add 250 μL of DMEM per well and keep the plate in cell culture incubator for up to 2 days. Do not dry the plate after matrigel coating.
7. Direct reprogramming of HFFs to pancreatic ductal-like cells:
  a. Day 0: Seed early-passage HFFs at 20,000 cells per well in matrigel-coated 24-well plate.
    ***Note:*** Scale the cell number based on the surface area if using other cell cultureware. Use the cells before passage 8.
  b. Day 1: Transduce with 6F pool with 8 μg/mL polybrene. Use multiplicity of infection (MOI) = 1 for SOX17, FOXA2 and PDX1; MOI = 2 for HNF1B, HNF6 and SOX9.
    ***Note:*** Although the cells are transduced with a pool of six TFs, cells carrying any sub-combinations of TFs from the 6F pool can also be present. This will result in the heterogeneity of reprogramming cells. Since ectopic TF-expressing cells can be identified based on their barcodes, the non-transduced cells without any barcodes can either be used as control cells or excluded from the analysis. Optionally, non-transduced cells can be eliminated during the experiment by a seven-day blasticidin selection at 5 μg/mL. The MOI used for each of the transduced TFs was determined based on their expression levels in ductal cells from adult human^11^ together with the earlier test results.^1^
  c. Day 2: Change to fresh fibroblast medium.
  d. Day 3: Change to basal medium for reprogramming cells supplemented with 100 ng/mL Activin A, 1 μM CHIR99021 and 50 ng/mL FGF7.
  e. Day 5: Change to basal medium for reprogramming cells supplemented with 100 ng/mL Activin A and 50 ng/mL FGF7.
  f. Day 7: Change to basal medium for reprogramming cells supplemented with 2 μM retinoic acid, 500 nM PD0325901 and 200 nM LDN193189.
  g. Day 8: Change to basal medium for reprogramming cells supplemented with 100 ng/mL Activin A and 500 nM PD0325901.
  h. From day 10 onwards, culture cells in maintenance medium for reprogramming cells. Pancreatic ductal-like cells with epithelial cell morphology start to appear at around day 21 (Figure 1C). Troubleshooting 4.
    ***Note:*** During cell reprogramming, passage cells at 1:2 ratio using Accutase when cells reach around 90% confluency and replate cells in fresh matrigel-coated plates.

### Preparing library for FI-snMultiome-seq analysis

**Timing: 3 days**

This section describes library preparation for FI-snMultiome-seq.

8. Collect reprogramming cells at desired time points and isolate nuclei using demonstrated protocol CG000365 from 10× Genomics. Troubleshooting 5 and 6.

**CRITICAL:** Using good quality cells is critical for the success of the experiments. Remove dead cells following the demonstrated protocol from 10× Genomics if cell viability is less than 90%. For balanced representation of different conditions in the analysis, isolate nuclei from individual conditions, count number of nuclei from each condition and pool the desired number of nuclei from different conditions for single-nuclei capture. Avoid pooling cells from different conditions before nuclei isolation.

***Note:*** The required number of nuclei to be profiled per condition depends on the experimental setting, e.g., the number of transduced TFs. For high quality (Grade A) nuclei, 800 nuclei per TF is a good starting point when overexpressing one TF. If the quality of nuclei is less optimal (Grade B), a minimum of 1,000 nuclei is recommend for a single-TF condition. Please refer to 10× Genomics instructions to assess the nuclei quality.

9. Prepare libraries for ATAC and GEX following user guide CG000338 from 10× Genomics.
10. Barcode library preparation for detecting TF barcodes:
  a. Prepare following reaction components on ice. Mix thoroughly and centrifuge briefly.

**Table:** PCR reaction master mix

Reagent	Amount
Pre-amplified sample	3 μL
KAPA HiFi HotStart ReadyMix	25 μL
10 μM Truseq_Read2_Vec_Amp_Forward Primer	2.5 μL
10 μM Truseq_Read1_Amp_Reverse Primer	2.5 μL
Nuclease-Free Water	to 50 μL

    ***Note:*** Pre-amplified sample is the purified product after step 4.3p from user guide CG000338.
  b. Start PCR using the cycling conditions described in the table below.

**Table:** PCR cycling conditions

Steps	Temperature	Time	Cycles
Initial denaturation	95°C	3 min	1
Denaturation	98°C	20 s	22 cycles
Annealing	65°C	15 s	
Extension	72°C	10 s	
Final extension	72°C	1 min	1
Hold	4°C	∞

    ***Note:*** To ensure the libraries are minimally amplified, add SYBR Green I to PCR reaction master mix and run a qPCR test. Calculate the optimal cycle number for each sample by determining the number of cycles required to reach 1/3 of the maximum R.
  c. Cleanup with SPRIselect beads:
    i. Vortex the SPRIselect beads until fully resuspended. Add 40 μL (0.8×) SPRIselect beads to each sample. Pipette mix 10 times.
    ii. Incubate 5 min at 20°C–25°C.
    iii. Centrifuge briefly. Place on the 10× magnetic separator (magnet·High) until the solution clears.
    iv. Remove the supernatant.
    v. Add 200 μL 80% ethanol to the pellet. Wait 30 s. Remove the ethanol. Repeat it.
    vi. Centrifuge briefly. Place on the magnet·Low. Remove any remaining ethanol.
    vii. Remove the tube strip from the magnet. Immediately add 40.5 μL Buffer EB.
    viii. Pipette mix 10 times and incubate 2 min at 20°C–25°C.
    ix. Centrifuge briefly. Place on the magnet·Low until the solution clears.
    x. Transfer 40 μL sample to a new tube strip.
      **Pause point:** Store at −20°C for long-term storage or proceed to the next step.
  d. Prepare following reaction components on ice. Mix thoroughly and centrifuge briefly.

**Table:** PCR reaction master mix

Reagent	Amount
Template DNA from step 10.c	50 ng
2× KAPA HiFi HotStart ReadyMix	25 μL
Dual Index TT Set A	10 μL
Nuclease-Free Water	to 50 μL

    **CRITICAL:** Choose different indices for the samples in a multiplexed sequencing run.
    ***Note:*** Barcode libraries can be pooled in the same sequencing run for GEX libraries if the indices are compatible.
  e. Start PCR using the cycling conditions described in the table below.

**Table:** PCR cycling conditions

Steps	Temperature	Time	Cycles
Initial denaturation	95°C	3 min	1
Denaturation	98°C	20 s	4 cycles
Annealing	65°C	15 s	
Extension	72°C	10 s	
Final extension	72°C	1 min	1
Hold	4°C	∞

    ***Note:*** To ensure the libraries are minimally amplified, add SYBR Green I to PCR reaction master mix and run a qPCR test. Calculate the optimal cycle number for each sample by determining the number of cycles required to reach 1/3 of the maximum R.
  f. Cleanup with SPRIselect beads:
    i. Vortex the SPRIselect beads until fully resuspended. Add 40 μL (0.8×) SPRIselect beads to each sample. Pipette mix 10 times.
    ii. Incubate 5 min at 20°C–25°C.
    iii. Centrifuge briefly. Place on the 10× magnetic separator (magnet·High) until the solution clears.
    iv. Remove the supernatant.
    v. Add 200 μL 80% ethanol to the pellet. Wait 30 s. Remove the ethanol. Repeat it.
    vi. Centrifuge briefly. Place on the magnet·Low. Remove any remaining ethanol.
    vii. Remove the tube strip from the magnet. Immediately add 30.5 μL Buffer EB.
    viii. Pipette mix and incubate 2 min at 20°C–25°C.
    ix. Centrifuge briefly. Place on the magnet·Low until the solution clears.
    x. Transfer 30 μL sample to a new tube strip.
      **Pause point:** Store the sample at −20°C for long term storage or proceed to the next step.
  g. Run 1 μL sample of ATAC, GEX and barcode libraries at 1:5 dilution on an Agilent TapeStation High Sensitivity D5000 ScreenTape to determine the average fragment size (Figures 2A–2C).

**Figure 2:**
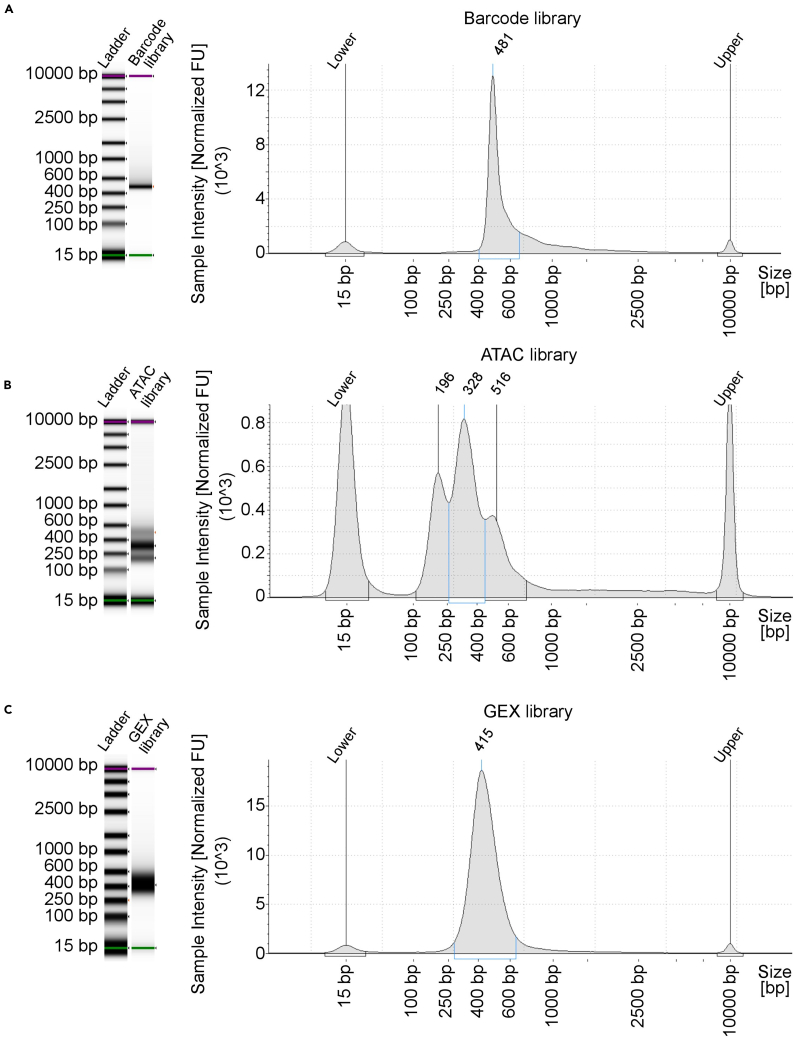
QC of final FI-snMultiome-seq libraries Tapestation trace of barcode library (A), ATAC library (B) and GEX library (C).
  h. Perform library quantification for sequencer clustering using KAPA Library Quantification Kit following manufacturer’s instructions and determine the concentration based on fragment size derived from TapeStation trace.
  i. Sequence ATAC and GEX libraries following user guide CG000338. Sequence barcode library using the same sequencing parameters for GEX library at a minimum of 2,000 read pairs per nucleus.
    ***Optional:*** Determine fragment size using Bioanalyzer or LabChip.

## Expected outcomes

The FI-snMultiome-seq protocol enables efficient capture of the TF expression from exogeneous expression vectors using a factor-indexing approach, which significantly improves the current 10× single cell multiome assay. This allows the identification and characterization of the cells that express all stochastic combinations of transduced TFs during direct reprogramming by reading the factor barcodes. Thus, it provides robust molecular analysis for comparing various reprogramming conditions and assessing the effect of individual TF in one experiment. Non-transduced cells can either be used as control or excluded from analysis to reduce the noise in the data. Notably, our methodology also enables segregating the expression of TFs from exogeneous vectors and endogenous genes by separately counting the transcripts from factor barcodes (exogeneous) and mRNA (endogenous). This allows the investigation of the effect of transduced TFs on its endogenous expression. Therefore, the application of FI-snMultiome-seq opens a path to study TF-mediated direct reprogramming at single-cell resolution, providing a comprehensive overview of the remodeling of transcriptomic and epigenomic landscape during transdifferentiation. Furthermore, the FI-snMultiome-seq protocol is versatile. The primers used for barcode insertion can easily be modified to work with other lentiviral constructs. Other than direct reprogramming, FI-snMultiome-seq can also be applied to other experimental conditions and cellular models that aim to study the cellular response to overexpression of genes from lentiviral constructs.

## Quantification and statistical analysis

The data were processed and analyzed as described in Fei et al.^1^ TF barcodes were extracted from the fastq files of the barcode library using the Sequence Input/Output interface (SeqIO)^4^ from BioPython^5^ and raw sequencing data from GEX and ATAC libraries were processed with Cell Ranger ARC pipeline (v2.0.1) for demultiplexing, alignment, and feature counting, followed by subsequent analysis using Seurat (v4.1.1)^6^ and Signac (v1.7.09).^7^ The number of reads for each TF barcode were counted and added as a column to the metadata of the Seurat object. Cells with <1,000 RNA unique molecular identifier (UMI) counts or ATAC fragments, >100,000 RNA UMI counts or >500,000 ATAC fragments, and >30% mitochondrial RNA were excluded. For scRNA-seq, the raw UMI counts were normalized and scaled using SCTransform, regressing out cell cycle effect and the effect of the percentage of the UMI counts originating from mitochondrial genes. The top 3,000 variable genes were selected for principal-component analysis (PCA). For scATAC-seq, peaks were called using MACS2 (v2.2.7.1).^8^ Peaks on nonstandard chromosomes and GRCh38 blacklist regions were excluded, followed by frequency inverse document frequency (TF-IDF) normalization and latent semantic indexing (LSI) reduction. The 1–35 PCs from the scRNA-seq data and the 2–35 LSI dimensions from the scATAC-seq data were used for constructing the weighted-nearest neighbor (WNN) graph. Per-cell motif activity scores were calculated using the Signac RunChromVAR wrapper.^9^ Cell type scores were computed using ScType (v1.0)^10^ with markers for pancreatic cell types from the ScType database and fibroblast markers from literature.^12^

## Limitations

FI-snMultiome-seq is based on lentiviral gene delivery. As with lentiviral expression systems, there could be potential silencing of viral expression after a few passages. This limits its application in analyzing mature reprogramming cells at late stages. Besides, cells carrying any sub-combinations from the TF pool can be present in theory when transducing a pool of TFs. However, it may happen that only some of the sub-combinations are present and the cell number of some conditions can be small. Thus, careful experimental planning and including additional cells for the sub-combination(s) of interest is important for optimal performance of FI-snMultiome-seq.

## Troubleshooting

### Problem 1

Low colony count after transformation (related to steps 1–2).

### Potential solution

• Confirm all the entry and destination clones by sequencing to check for the integrity of the BP and LR recombination sites.
• Confirm ampicillin concentration.
• Avoid multiple freeze-thaws of ligation product.
• Repeat transformation using a new fresh batch of competent cells.
• Purify the restriction digested product to remove contaminants and repeat the ligation.
• Purify the barcoding PCR product to remove contaminants. Repeat the restriction digest and ligation.

### Problem 2

Incorrect recombination of barcoded lentiviral vectors (related to step 3).

### Potential solution

• Always use Stbl3 competent cells or others that are suitable for propagation of lentiviral constructs.
• Grow the bacterial culture at lower temperature such as 30°C.
• Lower rotation speed to 200 rpm.
• Use the dual combination of ampicillin and chloramphenicol for selection.

### Problem 3

Low yield of lentiviruses (related to step 5).

### Potential solution

• Use early-passage 293FT cells.
• Avoid using overconfluent cells for viral production.
• Use fresh cell culture media.
• Harvest viruses twice. Collect viral supernatant on Day 3, add 20 mL of fresh complete medium per flask and do a second collection on Day 4. Combine the viruses from two collections.

### Problem 4

No morphological changes during reprogramming (related to step 7).

### Potential solution

• Use early-passage HFFs for reprogramming, no later than passage 8.
• Confirm the recipes of reprogramming medium.
• Avoid using expired supplements or medium.
• Avoid exposing the cells to 20°C–25°C for too long while passaging cells and taking microscope images.

### Problem 5

Low cell number of nuclei (related to step 8).

### Potential solution

We recommend starting with more than 500,000 reprogramming cells for nuclei isolation.

### Problem 6

Clumps and cell debris during nuclei isolation (related to step 8).

### Potential solution

• Wash cells with DPBS + 0.04% BSA for a total of 3 times before lysis to remove debris.
• Optimize cell lysis to obtain nuclei in good quality.
• Filter nuclei solution using 40 μm Flowmi Cell Strainer to remove large clumps.

## Resource availability

### Lead contact

Further information and requests for resources and reagents should be directed to and will be fulfilled by the lead contact, Biswajyoti Sahu (biswajyoti.sahu@ncmm.uio.no).

### Technical contact

Technical questions on executing this protocol should be directed to and will be answered by the technical contact, Liangru Fei (liangru.fei@ncmm.uio.no).

### Materials availability

This study did not generate new unique reagents.

### Data and code availability

This study did not generate new datasets. The code for extracting the TF barcode^1^ is available at GiHub: https://github.com/KaiyangZ/TF_barcode_extraction.
